# Design of Reconfigurable Time-to-Digital Converter Based on Cascaded Time Interpolators for Electrical Impedance Spectroscopy ^†^

**DOI:** 10.3390/s20071889

**Published:** 2020-03-29

**Authors:** Sounghun Shin, Yoontae Jung, Soon-Jae Kweon, Eunseok Lee, Jeong-Ho Park, Jinuk Kim, Hyung-Joun Yoo, Minkyu Je

**Affiliations:** 1 Foundry Business, Samsung Electronics Co., Ltd., Hwaseong-si, Gyeonggi-do 18448, Korea; sh01.shin@samsung.com; 2 School of Electrical Engineering, Korea Advanced Institute of Science and Technology (KAIST), Daejeon 34141, Korea; yoontae.jung@kaist.ac.kr (Y.J.); eunseoklee@kaist.ac.kr (E.L.); ray1206@kaist.ac.kr (J.K.); hjyoo53@kaist.ac.kr (H.-J.Y.); mkje@kaist.ac.kr (M.J.); 3 System LSI Business, Samsung Electronics Co., Ltd., Hwaseong-si, Gyeonggi-do 18448, Korea; jeongho.park@samsung.com

**Keywords:** electrical impedance spectroscopy (EIS), time-to-digital converter (TDC), time interpolator, phase, polar demodulator, quantization, reconfigurability

## Abstract

This paper presents a reconfigurable time-to-digital converter (TDC) used to quantize the phase of the impedance in electrical impedance spectroscopy (EIS). The TDC in the EIS system must handle a wide input-time range for analysis in the low-frequency range and have a high resolution for analysis in the high-frequency range. The proposed TDC adopts a coarse counter to support a wide input-time range and cascaded time interpolators to improve the time resolution in the high-frequency analysis without increasing the counting clock speed. When the same large interpolation factor is adopted, the cascaded time interpolators have shorter measurement time and smaller chip area than a single-stage time interpolator. A reconfigurable time interpolation factor is adopted to maintain the phase resolution with reasonable measurement time. The fabricated TDC has a peak-to-peak phase error of less than 0.72° over the input frequency range from 1 kHz to 512 kHz and the phase error of less than 2.70° when the range is extended to 2.048 MHz, which demonstrates a competitive performance when compared with previously reported designs.

## 1. Introduction

Electrical impedance spectroscopy (EIS), which measures the impedance over a range of frequencies, has been widely used in today’s biomedical applications such as body composition analysis [1,2,3], cancer diagnosis [4,5,6], and detection of allergic contact reaction [7], and so on. The frequency range from 1 kHz to several MHz, which is associated with the polarization of macromolecules [8], is required for these applications. With growing demands on portable EIS systems, today’s research is focused on designing fully integrated EIS systems that support a wide frequency range. 

The impedance spectrum can be obtained by measuring the impedance for a particular frequency and then repeating the measurement with sweeping the frequency, which is called the frequency response analyzing (FRA) method [9]. When a sinusoidal current signal is injected into the material under the experiment, the resulting sinusoidal voltage signal is generated with the magnitude and time delay that depend on the magnitude and phase of the material’s impedance, respectively [10,11]. The EIS system is generally composed of two parts: a sinusoidal signal generator (SSG) and a demodulator. The SSG injects the sinusoidal current signal into the target material, and the demodulator measures the resulting voltage signal. Considering that the EIS system that supports sensor array includes several demodulators with one SSG [9,12,13], it is important to design a demodulator to be hardware-efficient and to have high precision.

Conventional quadrature demodulators extract real and imaginary parts of impedance using quadrature mixers and low-pass filters (LPFs) [12,13,14]. When the resulting signal depending on the target impedance is multiplied by a signal that is in phase with the injected signal and then low-pass filtered, the output value represents the real part of the impedance. When multiplied by a signal that is 90° out of phase with the injected signal and then low-pass filtered, the imaginary part of the impedance is provided. In such a process, incomplete synchronization among the aforementioned signals becomes a dominant source of error, and the need for phase-correction circuitry increases the implementation complexity [15]. To resolve this synchronization issue, polar demodulators, which asynchronously measure the magnitude variation and time delay of the resulting signal, have been proposed [10,11,15,16,17]. In particular, the polar demodulators in [10,11,17] mitigate the complexity of analog front-end significantly because the magnitude variation is measured without large-time-constant LPFs and the time delay is measured with one of simple logics such as XOR, XNOR, and latch. In quadrature demodulators, the large-time-constant LPFs limit measurement speed [17] and occupy large area. In polar demodulators, contrarily, the simple logic produces a pulse having the width that corresponds to the time delay, and then a time-to-digital converter (TDC) is used to quantize the pulse width. Note that the pulse width is proportional not only to the phase of impedance but also to the period of the injected signal. Therefore, the TDC should be able to handle wide input-time range to support the analysis at low frequencies, and also achieve a high resolution to support the analysis at high frequencies. It is, therefore, important to design the TDC to meet these requirements in a simple and efficient way for implementing a low-complexity polar demodulator.

Two types of TDCs have been proposed for the EIS system: TDCs based on a time-to-voltage converter (TVC) [15] and a counter [17,18,19]. The TVC converts the pulse width to the voltage by charging a capacitor with a current source, and the following analog-to-digital converter (ADC) quantizes the output of the TVC [15]. However, the phase error increases with the frequency of the injected signal because the full-scale output voltage of the TVC decreases as the frequency of the injected signal increases. Since counter-based TDCs have an input-time range that is theoretically unlimited [20], this type of TDCs appear to be attractive phase quantizers in the EIS system. However, the TDC in [17] requires high-speed counting clock for high-frequency analysis because the counting speed solely decides the time resolution. This TDC adopts a 3.3-GHz counting clock to support the maximum frequency of up to 10 MHz.

We have proposed two reconfigurable TDCs combining counters with time interpolators to improve resolution without increasing the counting clock speed and verified them by simulation [18,19]. Both TDCs employ a coarse counter to secure wide input-time range. The TDC in [18] uses cascaded time interpolators with a reconfigurable time interpolation factor. When the same large interpolation factor is used, the cascaded time interpolators have shorter measurement time and smaller chip area than a single-stage time interpolator. Since both the required input-time range and time resolution vary with the frequency of the injected signal, the reconfigurable time interpolation factor is employed to maintain phase error within an appropriate level while providing reasonable measurement time. Instead of the cascaded time interpolators, the TDC in [19] utilizes a time interpolator with a low interpolation factor and a chain delay line to further reduce the phase error. Although the TDC in [19] shows improved precision, the TDC in [18] allows more efficient implementation in terms of chip size.

This paper presents the reconfigurable TDC based on cascaded time interpolators [18], which has been fabricated in 0.25-μm CMOS process, with providing comprehensive explanation, detailed analysis, and measurement results. The fabricated TDC quantizes the pulse width corresponding to the phase of 0° to 90° with the peak-to-peak phase error of under 0.72° up to 512-kHz frequency and the phase error of under 2.70° up to 2.048-MHz frequency, demonstrating competitive performances compared to previously reported designs. 

## 2. Background and Design Specifications

This section describes the impedance measurement principle and phase measurement scheme in EIS systems. From this background, design specifications are derived at the end of the section.

### 2.1. Impedance Measurement Principle

**Figure 1:**
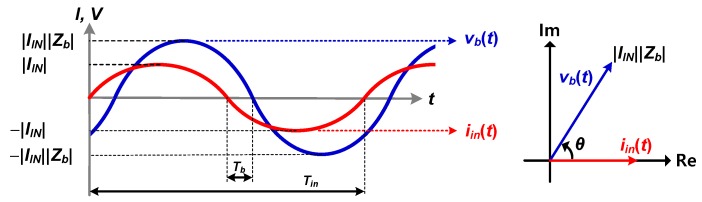
Magnitude and phase of the resulting voltage determined by the impedance under measurement.

When a sinusoidal current signal *i_in_*(*t*) is injected into the target material, a resulting sinusoidal voltage signal *v_b_*(*t*) is generated, and its magnitude and time delay with respect to *i_in_*(*t*) depend on the impedance of the material, as shown in Figure 1. *Z_b_* is the impedance of the target material, and |*Z_b_*| and *θ* are the magnitude and phase of *Z_b_*, respectively. 

Polar demodulators measure the magnitude and time delay of *v_b_*(*t*) for a particular frequency. |*Z_b_*| and *θ* can be calculated as follows:

(1) $$\left|Z_{b}\right|=\frac{\left|v_{b}\left(t\right)\right|}{\left|I_{IN}\right|},$$

(2) $$\theta =\frac{T_{b}}{T_{in}},$$

where |*v_b_*(*t*)| and *T_b_* are the magnitude and time delay of *v_b_*(*t*), respectively. |*I_IN_*| is the magnitude of *i_in_*(*t*), and *T_in_* is the period of *i_in_*(*t*) and *v_b_*(*t*). Impedance spectrum is obtained by using the FRA method, which analyzes one frequency at a time, repeatedly with sweeping the frequency [9]. 

### 2.2. Phase Measurement Scheme in Polar Demodulators

**Figure 2:**
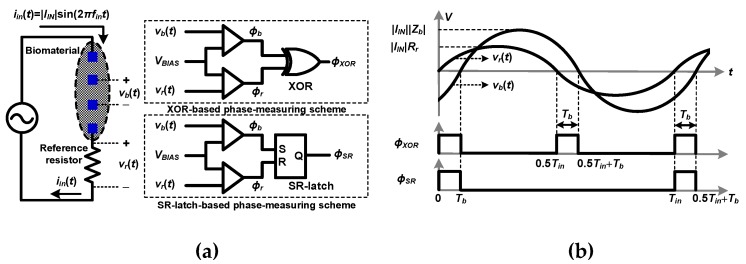
(**a**) Block diagram and (**b**) waveforms of the phase measurement scheme (reproduced from [19] with permission from the IEEE).

Figure 2 shows the phase measurement scheme adopted in polar demodulators [10,11,15,16,17]. When *i_in_*(*t*) is injected into the target material and reference resistor, *v_r_*(*t*) with the same phase as *i_in_*(*t*) is generated from the resistor in addition to *v_b_*(*t*). Comparators convert *v_r_*(*t*) and *v_b_*(*t*) to clock signals, *ϕ_r_* and *ϕ_b_*, from which an XOR gate and an SR latch create clock signals, *ϕ_XOR_* and *ϕ_SR_*, respectively. These clock signals have the pulse width of *T_b_*, which corresponds to *θ*, as shown in Figure 2. From Equation (2), when the frequency of the injected current, *f_in_* = 1/*T_in_* is known, *θ* can be determined by measuring *T_b_*.

### 2.3. Design Specifications

Design specifications of TDC proposed in this paper are presented in Table 1. *f_in_* is set from 1 kHz to 2.048 MHz because the proposed TDC is implemented in the form of a fully integrated chip for EIS systems in biomedical applications. Considering that the impedance measured in such applications [15] usually has capacitive reactance, *θ* ranges from 0° to 90°, and the corresponding input-time range is 0 to 122 ns at the shortest when *f_in_* is 2.048 MHz and 0 to 250 μs at the widest when *f_in_* is 1 kHz. Referring to performances of the previous works reported in [15,17], this TDC aims to have maximum phase error under 1° and phase resolution over 10 bits for the suggested range of *f_in_*. This amount of phase error corresponds to 1.35 ns in the worst case when *f_in_* is 2.048 MHz. Lastly, the frequency of the reference clock (*f_clk_*) is set to 32.768 MHz, which is only 16 times the maximum *f_in_*. Compared to the TDC in [17], where *f_clk_* is 330 times higher than the maximum *f_in_*, the proposed TDC aims to achieve competitive phase error performance with much lower *f_clk_*.

## 3. Architecture of the Proposed TDC

As shown in Table 1, the requirements of both the input-time range and time resolution vary with *f_in_*. The proposed TDC operates across three different modes to meet the design specifications with maintaining reasonable measurement time. This section describes overall architecture and operation in each mode. 

### 3.1. Overall Architecture and Operation

**Figure 3:**
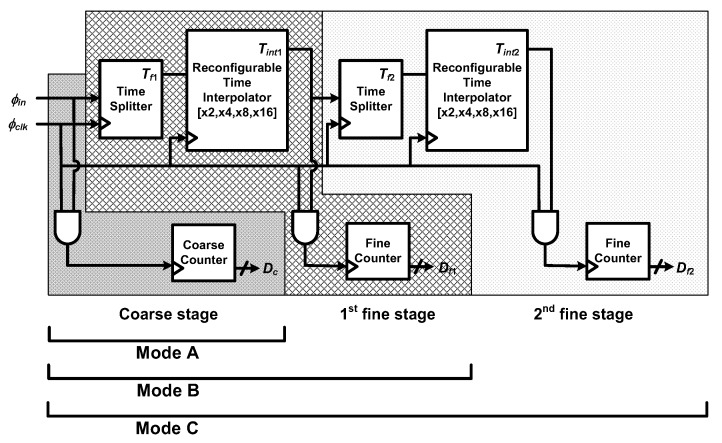
Block diagram of the proposed TDC (reproduced from [18] with permission from the IEEE).

The block diagram of the proposed TDC is presented in Figure 3. The input pulse signal whose pulse width carries *θ* is denoted as *ϕ_in_*. *ϕ_clk_* is the reference clock, and the outputs of the system are the digital bits, *D_c_*, *D_f_*_1_, and *D_f_*_2_. This TDC is composed of three stages, namely a coarse stage, the first fine stage, and the second fine stage. The coarse stage consists of a 12-bit coarse counter with digital logics. Each fine stage consists of a time splitter, a reconfigurable time interpolator, and a 4-bit counter. The coarse counter is used to implement a wide input-time range, and two fine stages are employed to improve resolution without increasing *f_clk_*. In each fine stage, the time splitter extracts quantization error of the preceding stage and the time interpolator stretches the quantization error. The resolution can be improved by quantizing the stretched quantization error through the fine counters and *ϕ_clk_*.

The proposed TDC operates in one of the three modes to achieve the phase resolution over 10 bits. In mode A, only the coarse stage is used, and the time resolution is *T_clk_*. For low *f_in_* of 1 kHz and 2 kHz, *f_clk_* is high enough to achieve target phase resolution. For 4-kHz *f_in_* upwards, the fine stages are used with the coarse stage to further improve the time resolution without increasing *f_clk_*. In mode B, the coarse stage and the first fine stage are used for *f_in_* from 4 kHz to 32 kHz. The first fine stage further quantizes the quantization error of the coarse stage with a time interpolation factor of *A_T_*_1_. The time resolution in mode B is *T_clk_*/*A_T_*_1_, which is *A_T_*_1_ times higher than the highest resolution in mode A while keeping *f_clk_* = 32.768 MHz. In mode C, for 32-kHz *f_in_* upwards, the coarse stage and the first fine stage operate in the same manner. In addition, the second fine stage further quantizes the quantization error of the first fine stage. The time resolution in mode C is *T_clk_*/(*A_T_*_1_*A_T_*_2_), which is *A_T_*_1_*A_T_*_2_ times higher than the highest one in mode A, still keeping *f_clk_* = 32.768 MHz. When *f_in_* = 2.048 MHz, the coarse stage only achieves a 2-bit resolution for *θ* range from 0° to 90° with *f_clk_* = 32.768 MHz. Therefore, the total interpolation factor (*A_T_*) of up to 256 is required to achieve a 10-bit resolution for the whole *f_in_* range. *A_T_*_1_ and *A_T_*_2_ are set, as shown in Table 2, across three different modes, A, B, and C, for varying values of *f_in_*. 

When utilizing the time interpolator, *A_T_* is determined by the ratio of discharging capacitance and discharging current [20]. If the current increases for implementing a large *A_T_*, the power consumption of TDC increases accordingly. Moreover, since the pulse width of the interpolated signal increases, the conversion time increases significantly, which leads to the degraded conversion rate. Thus, the large *A_T_* is realized in two steps by dividing *A_T_* into *A_T_*_1_ and *A_T_*_2_ to offer much more relaxed design conditions. *A_T_*_1_ and *A_T_*_2_ can be adjusted between 2, 4, 8, and 16 to provide 1, 2, 3, and 4 additional bits, respectively. Reconfigurable *A_T_*_1_ and *A_T_*_2_ maintain the phase resolution over 10 bits with reasonably short measurement time.

### 3.2. Operation in Mode A

**Figure 4:**
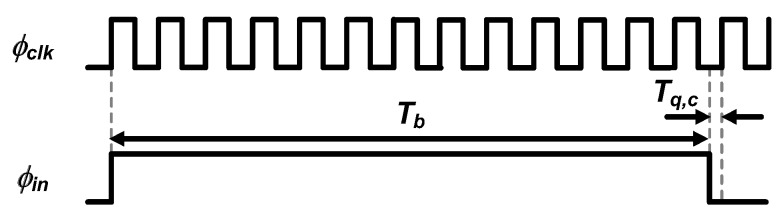
Timing diagram of the proposed TDC in mode A.

This mode offers only counter-based time quantization, and the timing diagram of its operation is depicted in Figure 4.

The proposed TDC in mode A outputs digital bits *D_c_* with the relation as follows:

(3) $$T_{b}=D_{c}\times T_{clk}-T_{q,c},$$

where *Τ_q,c_* is the quantization error of the coarse stage and smaller than *Τ_clk_*. The time resolution in mode A becomes one period of the clock signal, *T_clk_*. To achieve a 12-bit phase resolution for *θ* range from 0° to 90° at low *f_in_*, this mode is used for *f_in_* of up to 2 kHz.

### 3.3. Operation in Mode B

**Figure 5:**
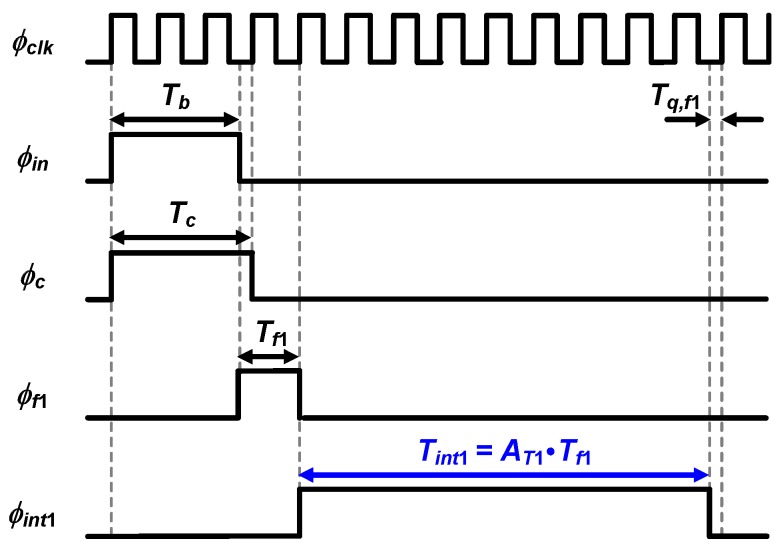
Timing diagram of the proposed TDC in Mode B.

Mode B uses the coarse stage and the first fine stage, each generating output digital bits, *D_c_* and *D_f_*_1_, respectively. The timing diagram of its operation is presented in Figure 5. 

The pulse width of *T_f_*_1_ is generated by the time splitter in the first fine stage and expressed as follows:

(4) $$T_{f1}=T_{q,c}+T_{clk}.$$

The time interpolator in the first stage stretches *T_f_*_1_ to *T_int_*_1_, which is described by:

(5) $$T_{\mathrm{int}1}=A_{T1}\cdot T_{f1}.$$

The fine counter quantizes *T_int_*_1_ with the reference clock, to output up to four fine digital bits, *D_f_*_1_. The relation among *T_b_*, *D_c_*, and *D_f_*_1_ is given by:

(6) $$T_{b}=T_{c}-\left(T_{f1}-T_{clk}\right)=\left(D_{c}\times T_{clk}\right)-\left(\frac{T_{\mathrm{int}1}}{A_{T1}}-T_{clk}\right)=\left(D_{c}\times T_{clk}\right)-\left(\frac{D_{f1}\times T_{clk}-T_{q,f1}}{A_{T1}}-T_{clk}\right),$$

where *Τ_q,f_*_1_/*A_T_*_1_ is always smaller than *Τ_clk_*. The time resolution in mode B is improved from *T_clk_* to *T_clk_*/*A_T_*_1_, which is *A_T_*_1_ times higher than the highest resolution in mode A while keeping *f_clk_* = 32.768 MHz. To achieve a 12-bit phase resolution for *θ* range from 0° to 90°, *f_in_* must satisfy the following condition:

(7) $$\frac{1}{f_{in}}\cdot \frac{90}{360}\cdot \frac{1}{2^{12}}<\frac{T_{clk}}{A_{T1}}.$$

With the maximum *A_T_*_1_ of 16, this mode is used until *f_in_* increases up to 32 kHz.

### 3.4. Operation in Mode C

**Figure 6:**
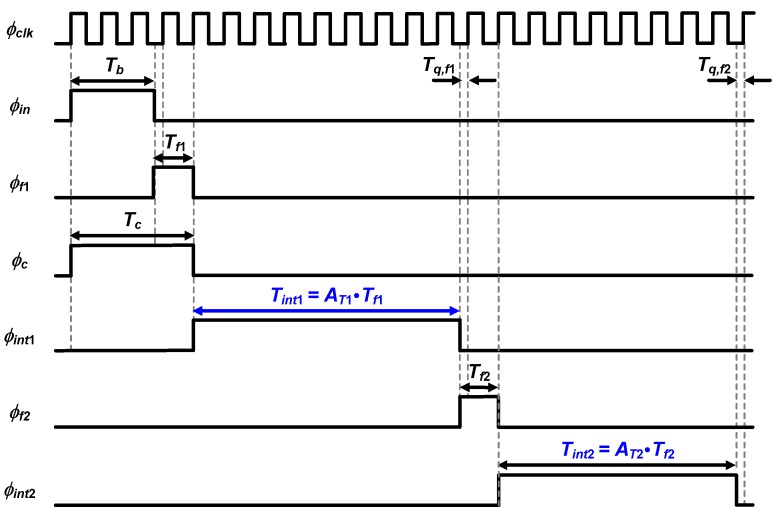
Timing diagram of the proposed TDC in mode C.

The mode C uses the coarse stage, the first fine stage, and the second fine stage, each generating output digital bits, *D_c_*, *D_f_*_1_, and *D_f2_*, respectively. The timing diagram of its operation is presented in Figure 6. 

The coarse stage and the first fine stage operate in the same manner as in mode B. The time splitter in the second stage generates *T_f_*_2_, which is expressed as:

(8) $$T_{f2}=T_{q,f1}+T_{clk}.$$

The time interpolator in the second stage stretches *T_f_*_2_ to *T_int_*_2_, which is described by:

(9) $$T_{\mathrm{int}2}=A_{T2}\cdot T_{f2}.$$

The fine counter in the second fine stage quantizes *T_int_*_2_ with the reference clock, to output up to four fine digital bits, *D_2_*. The relation among *T_b_*, *D_c_*, *D_f_*_1_, and *D_f_*_2_ is given by:

(10) $$\begin{array}{ll} T_{b} & =\left(D_{c}\times T_{clk}\right)-\left(\frac{D_{f1}\times T_{clk}-T_{q,f1}}{A_{T1}}-T_{clk}\right)=\left(D_{c}\times T_{clk}\right)-\left\{\frac{D_{f1}\times T_{clk}-\left(T_{f2}-T_{clk}\right)}{A_{T1}}-T_{clk}\right\}\\ & =\left(D_{c}\times T_{clk}\right)-\left\{\frac{D_{f1}\times T_{clk}-\left(\frac{D_{f2}\times T_{clk}-T_{q,f2}}{A_{T2}}-T_{clk}\right)}{A_{T1}}-T_{clk}\right\}\\ & =\left\{\left(D_{c}+1\right)\times T_{clk}\right\}-\left\{\frac{\left(D_{f1}-1\right)\times T_{clk}}{A_{T1}}\right\}+\left(\frac{D_{f2}\times T_{clk}-T_{q,f2}}{A_{T1}A_{T2}}\right), \end{array}$$

where *Τ_q,f_*_2_/(*A_T_*_1_*A_T_*_2_) is always smaller than *Τ_clk_*. The time resolution in mode C is improved from *T_clk_* to *T_clk_*/(*A_T_*_1_*A_T_*_2_), which is *A_T_*_1_*A_T_*_2_ times higher than the highest resolution in mode A, even though *f_clk_* = 32.768 MHz is kept. To achieve a 10-bit phase resolution for *θ* range from 0° to 90°, *f_in_* must satisfy the following condition:

(11) $$\frac{1}{f_{in}}\cdot \frac{90}{360}\cdot \frac{1}{2^{10}}<\frac{T_{clk}}{A_{T1}A_{T2}}.$$

with the maximum *A_T_*_1_ and *A_T_*_2_ of 16, this mode is used for *f_in_* of up to 2.048 MHz.

## 4. Circuit Design

This section describes how the first fine stage is designed to realize time interpolation with reconfigurable *A_T_*_1_. The second fine stage is designed to be identical to the first one. 

### 4.1. Time Splitter

**Figure 7:**
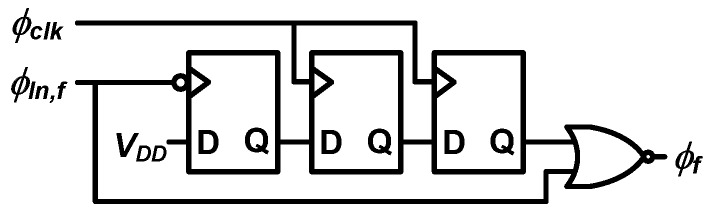
Structure of the time splitter (reproduced from [18] with permission from the IEEE).

Figure 7 shows the structure of the time splitter, which consists of three D flip-flops and one NOR gate. As shown in Figure 5 and Figure 6, the time splitter extracts the quantization error of the coarse stage with an offset of *T_clk_*. This offset is employed to avoid the metastability issue of the D flip-flops [20]. Therefore, *T_f_*_1_ takes the value from *T_clk_* to 2*T_clk_*, corresponding to 30.52 ns to 61.04 ns. As shown in Equations (6) and (10), in mode B and C, the offset is compensated when *T_b_* is calculated from the digital outputs.

### 4.2. Reconfigurable Time Interpolator

**Figure 8:**
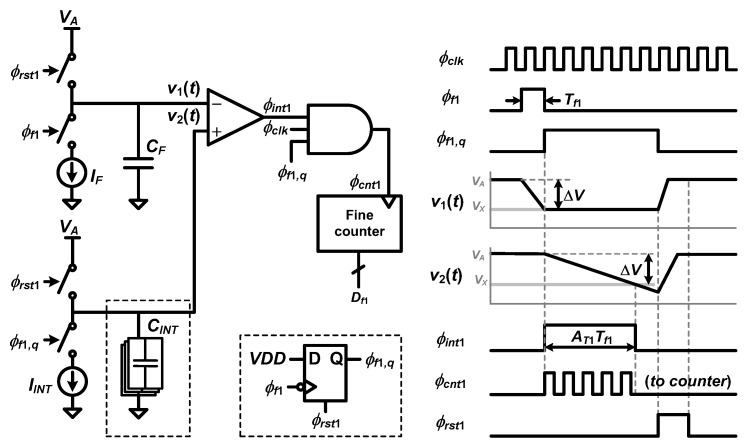
Block diagram and timing diagram of the reconfigurable time interpolator (reproduced from [18] with permission from the IEEE).

Figure 8 shows the block diagram and timing diagram of the reconfigurable time interpolator. The reconfigurable time interpolator is similar to the time interpolator in [20]. A variable discharging capacitor, which has the capacitance of *C_INT_*, is added to obtain reconfigurable *A_T_*_1_. 

While *ϕ_f_*_1_, which has the pulse width of *T_f_*_1_, is high, a capacitor which has the capacitance of *C_F_* is discharged by a constant current, *I_F_*, such that *v*_1_(*t*) drops by Δ*V*. Δ*V* is expressed as follows:

(12) $$\Delta V=\frac{I_{F}\cdot T_{f1}}{C_{F}}.$$

From the falling edge of *ϕ_f_*_1_, a capacitor of *C_INT_* is discharged by a constant current, *I_INT_*, during *ϕ_f_*_1*,q*_ is high. In the same manner with Equation (12), the pulse width of *ϕ_f_*_1*,q*_ is expressed as follows:

(13) $$A_{T1}\cdot T_{f1}=\frac{C_{INT}}{I_{INT}}\cdot \Delta V=\frac{C_{INT}}{I_{INT}}\cdot \frac{I_{F}}{C_{F}}\cdot T_{f1}=\frac{C_{INT}}{C_{F}}\cdot \frac{I_{F}}{I_{INT}}\cdot T_{f1}=\left(M\cdot N\right)\cdot T_{f1},$$

where *M* is the capacitance ratio, *C_INT_*/*C_F_*, *N* is the current ratio, *I_F_*/*I_INT_*, and *A_T_*_1_ is *M·N*. 

In the proposed TDC, *N* is kept constant while *M* is controlled to change *A_T_*_1_ between 2, 4, 8, and 16. Adjustment of the capacitance value is selected over the current value because controlling the capacitance ratio is more accurate than controlling the current ratio in IC implementation. In each fine stage, since *N* is fixed to 2, *C_F_* is kept constant as 3.6 pF, and *C_INT_* is changed across 3.6 pF, 7.2 pF, 14.4 pF, and 28.8 pF. *I_F_* and *I_INT_* are 80 μA and 40 μA, respectively. 

### 4.3. Novel Features of the Proposed TDC

The proposed TDC employs time interpolation technique, which can improve time resolution without increasing *f_clk_* in the counter-based TDC. However, two inherent issues are associated with large *A_T_*. Although the resolution becomes much higher when the front-stage quantization error is interpolated with *A_T_*, the chip size or power consumption increases by a substantial amount because *A_T_* is determined by capacitance ratio or current ratio. Also, the conversion time increases significantly because the interpolated pulse width increases as *A_T_* increases. A novel structure of cascading two separate fine stages resolves these two issues at the same time.

When *A_T_* of 256 is obtained by using a single-stage time interpolator with *C_F_* of 1 pF and *C_INT_* of 256 pF, this results in excessively large chip size and poor area efficiency. In the proposed TDC, two interpolation stages are cascaded. As a result, the interpolation factor of only 16 is required in each stage instead of 256. In other words, this system requires two capacitors with the size of *C_INT_*, which is equal to 16 *C_F_*. Compared to the single stage with *A_T_* of 256, this approach reduces the area used for implementing the discharging capacitors by a factor of 257/34 = ~7.6 times considering that one time interpolator has two discharging capacitors with the sizes of *C_INT_* and *C_F_*. As the time interpolators in fine stages occupy a significant portion of the chip size, the area efficiency of the system is greatly improved. On the other hand, when *A_T_* of 256 is set by the capacitance ratio, the current consumption of the cascaded time interpolation stages is two times higher than that of the single-stage time interpolator. For the single interpolator with *A_T_* of 256, from the falling edge of *ϕ_c_*, the conversion time of *A_T_T_f_*_1_ is required for fine conversion, and the maximum conversion time is 2*A_T_T_clk_* considering the offset of the time splitter. For two cascaded interpolation stages with *A_T_*_1_ and *A_T_*_2_ of 16, the conversion time of each fine stage is *A_T_*_1_*T_f_*_1_ or *A_T_*_2_*T_f_*_1_, and the maximum conversion time of each stage is 2*A_T_*_1_*T_clk_* or 2*A_T_*_2_*T_clk_*. Compared to the single interpolator with *A_T_* of 256, when *A_T_*_1_ = *A_T_*_2_ = 16, the conversion time for fine conversion is reduced by approximately 8 times. 

When *N_f_* of time interpolation stages are cascaded and *A_T_* is set by the capacitance ratio, the area efficiency (*E_A_*), power efficiency (*E_P_*), and conversion-time efficiency for fine stages (*E_C_*) can be defined and expressed as follows:

(14) $$E_{A}=\frac{\mathrm{Total}\;\mathrm{capacitance}\;\mathrm{in}\;\mathrm{an}\;\mathrm{interpolation}\;\mathrm{stage}\;\mathrm{when}\;N_{f}=1\;}{\mathrm{Total}\;\mathrm{capacitance}\;\mathrm{in}\;\mathrm{interpolation}\;\mathrm{stages}\;\mathrm{when}\;N_{f}>1\;}=\frac{\left(1+A_{T}\right)C_{F}}{N_{f}\left(1+\sqrt[{N_{f}}]{A_{T}}\right)C_{F}}=\frac{1+A_{T}}{N_{f}\left(1+\sqrt[{N_{f}}]{A_{T}}\right)},$$

(15) $$E_{P}=\frac{\mathrm{The}\;\mathrm{current}\;\mathrm{consumption}\;\mathrm{of}\;\mathrm{an}\;\mathrm{interpolation}\;\mathrm{stage}\;\mathrm{when}\;N_{f}=1}{\mathrm{The}\;\mathrm{current}\;\mathrm{consumption}\;\mathrm{of}\;\mathrm{interpolation}\;\mathrm{stages}\;\mathrm{when}\;N_{f}>1}=\frac{I_{fine}}{N_{f}\cdot I_{fine}}=\frac{1}{N_{f}},$$

(16) $$E_{C}=\frac{\mathrm{The}\;\max.\;\mathrm{conversion}\;\mathrm{time}\;\mathrm{through}\;a\;\mathrm{fine}\;\mathrm{stage}\;\mathrm{when}\;N_{f}=1}{\mathrm{The}\;\max.\;\mathrm{conversion}\;\mathrm{time}\;\mathrm{through}\;\mathrm{fine}\;\mathrm{stages}\;\mathrm{when}\;N_{f}>1\;}\approx \frac{A_{T}\cdot 2T_{clk}}{N_{f}\cdot \sqrt[{N_{f}}]{A_{T}}\cdot 2T_{clk}}=\frac{A_{T}}{N_{f}\cdot \sqrt[{N_{f}}]{A_{T}}},$$

where *I_fine_* is the current consumption of a single time interpolation stage.

Table 3 summarizes *E_A_*, *E_P_*, and *E_C_* calculated using Equations (14)–(16). Although the maximum *E_A_*·*E_P_*·*E_C_* value is obtained when *N_f_* = 4, we chose *N_f_* = 2 to minimize the current consumption while taking advantages of the cascaded time interpolators in terms of area and conversion time. The largest capacitor with size of 28.8 pF is small enough to integrate on chip and 2*A_T_*_1_*T_clk_* = ~1 μs of conversion time for the fine stage is short enough.

It is also possible to obtain *A_T_* through the current ratio of *I_F_*/*I_INT_* in Figure 8. In this case, setting the current ratio to 256 directly affects the static power, severely degrading the power efficiency of the system. Total discharging current of cascaded time interpolation stages when *N_f_* = 2 could be 7.6-times smaller than that of a single time interpolation stage when *N_f_* = 1, and the total capacitance of cascaded time interpolation stages when *N_f_* = 2 could be two times larger than that of a single time interpolation stage when *N_f_* = 1. Compared to the single interpolator with *A_T_* of 256 and *N_f_* = 1, when *N_f_* = 2, the time for fine conversion is reduced by approximately eight times. The optimization process when *A_T_* is set by the current ratio would be similar to that when *A_T_* is set by the capacitance ratio. 

## 5. Measurement Results

**Figure 9:**
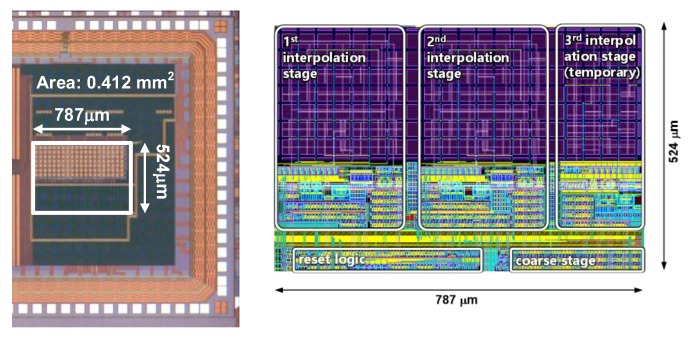
Chip photograph and layout.

The proposed TDC has been fabricated with a 0.25-μm CMOS process. The size of the circuit is 787 μm × 524 μm (0.412 mm^2^). The chip photograph and layout of the fabricated IC are presented in Figure 9.

**Figure 10:**
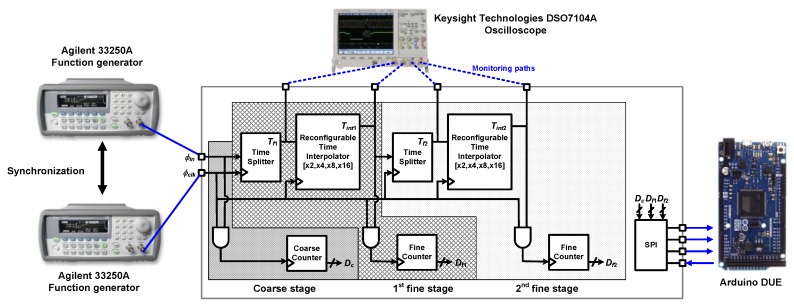
Measurement setup.

Figure 10 shows the measurement setup. Two Agilent 33250A function generators are used, one for generating *ϕ_clk_* and another for generating *ϕ_in_* whose pulse width varies from 0 to 0.25 of the period which corresponds to the phase of 0° to 90°. These two function generators are synchronized with each other. In the case of generating *ϕ_in_* in the form of pulse train, the period can be adjusted from 20.00 ns to 2000.0 s, and its pulse width can be controlled from 8.0 ns to 1999.9 s. Therefore, when *f_in_* = 2.048 MHz, the phase above 5.9° could be measured. Arduino DUE was selected for a microcontroller unit because it has 54 digital I/O pins which are enough for receiving digital output bits and transmitting assignment codes through SPI for the reconfiguration of TDC. Moreover, its clock speed of 84 MHz allows generating required signals to initialize the SPI communication and select between the read and write modes. Monitoring pads are placed on the main propagation path of signal to examine whether each block and each stage operate as expected. Keysight Technologies DSO7104A oscilloscope was used throughout the measurement. The oscilloscope has enough sample rate of 4 Gsps, bandwidth of 1 GHz, and four scope channels.

**Figure 11:**
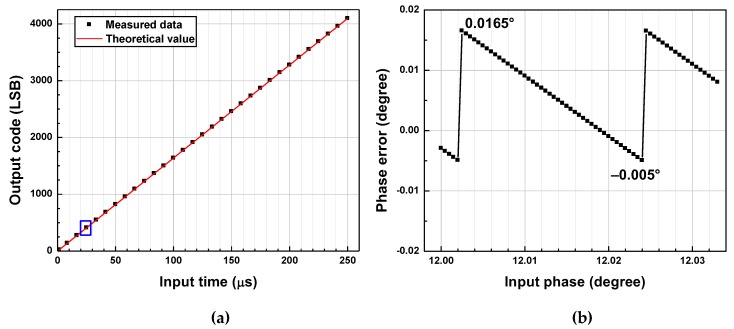
(**a**) Measured input-output characteristic of TDC, and (**b**) phase errors for *f_in_* = 1 kHz.

**Figure 12:**
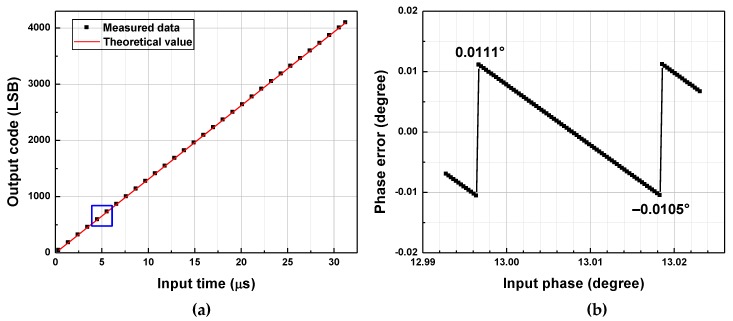
(**a**) Measured input-output characteristic of TDC, and (**b**) phase errors for *f_in_* = 8 kHz.

**Figure 13:**
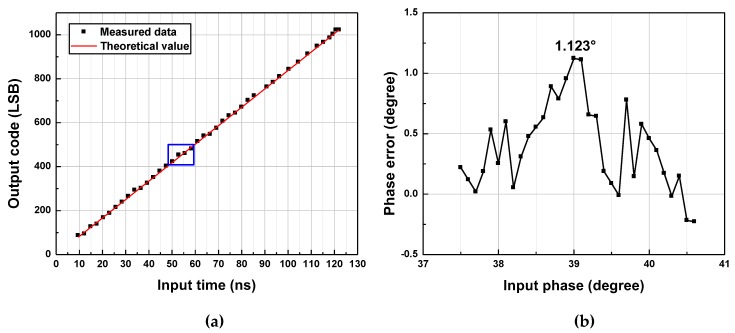
(**a**) Measured input-output characteristic of TDC, and (**b**) the maximum positive phase error for *f_in_* = 2.048 MHz.

In this section, measurement results for one frequency in mode A, one in mode B, and one in mode C are shown. The input-output characteristics of the fabricated TDC are described in Figure 11a, Figure 12a and Figure 13a. Figure 11b, Figure 12b and Figure 13b present the phase errors in each frequency.

### 5.1. Mode A

Measurement results for *f_in_* = 1 kHz are shown in Figure 11. Only the coarse stage is used, and the TDC operates as a counter-based TDC with *f_clk_* = 16.384 MHz, halved from the 32.768-MHz clock signal provided by the function generator. The input-output characteristic of the TDC is shown in Figure 11a, where the horizontal axis indicates the pulse width of the input signal in seconds, and the vertical axis shows the TDC output code in units of LSB. The solid line represents theoretical output values for given pulse widths of the input. Figure 11b presents the phase error calculated from the digital output code as a function of the actual phase injected at the input. The input phase is varied with a step size of 0.0005°, which corresponds to about 1.4 ns. The difference between the maximum and minimum phase errors is about 0.022°, implying that the error of the measured output lies within 12-bit-resolution quantization error for *θ* range from 0° to 90° and that the TDC operates as expected.

### 5.2. Mode B

Measurement results for *f_in_* = 8 kHz are shown in Figure 12. The coarse stage and the first fine stage are used with *f_clk_* = 32.768 MHz and *A_T_*_1_ = 4. However, *A_T_*_1_ of 4.05 is obtained from the measured *T_int_*_1_ and *T_f_*_1_, and this value is substituted to Equation (6) to derive *T_b_* using output code. The input-output characteristic of the TDC is shown in Figure 12a, where the horizontal axis, vertical axis, and solid line are as described in Figure 11a. Figure 12b presents the phase error calculated from the digital output code as a function of the actual phase injected at the input. The input phase is varied with a step size of 0.0003°, which corresponds to 0.1 ns. The peak-to-peak phase error is about 0.022°, implying that the error of the measured output lies within 12-bit-resolution quantization error for *θ* range from 0° to 90°. This result shows a good agreement with the theoretical prediction.

### 5.3. Mode C

Measurement results for *f_in_* = 2.048 MHz are shown in Figure 13. For *f_in_* = 2.048 MHz, *A_T_*_1_ and *A_T_*_2_ are both set to 16 to achieve the largest *A_T_* of 256. However, *A_T_*_1_ of 15.9 and *A_T_*_2_ of 15.9 are obtained, and these values are substituted to Equation (10) to derive *T_b_* using output code. The input-output characteristic of the TDC is shown in Figure 13a, where the horizontal axis, vertical axis, and the solid line are as described in Figure 11a. Figure 13b presents the phase error calculated from the digital output code as a function of the actual phase injected at the input. The input phase is varied with a step size of 0.07°, which corresponds to 0.1 ns. The peak positive phase error is 1.123°, and the peak negative phase error is −1.846°, resulting in the peak-to-peak phase error of 2.969°. This phase error is beyond the target quantization error of 0.088° and target phase error of 1°. The error analysis for the mode-C operation is presented in the next sub-section with a summary of the measurement results.

### 5.4. Error Analysis

**Figure 14:**
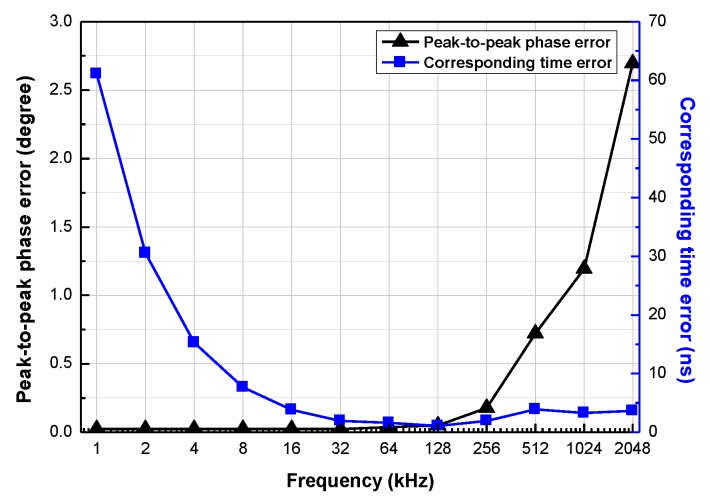
Peak-to-peak phase error and corresponding time error for *f_in_* from 1 kHz to 2.048 MHz.

The peak-to-peak phase error and corresponding time error for *f_in_* from 1 kHz to 2.048 MHz are described in Figure 14. The peak-to-peak phase error is proportional to *f_in_* and exceeds 1° for only when *f_in_* = 1.024 MHz and *f_in_* = 2.048 MHz. 

Table 4 summarizes the exact values of the peak-to-peak phase error and corresponding time error as a function of *f_in_*. It also shows the values of *A_T_*_1_ and *A_T_*_2_ set for the measurement. Although *A_T_*_2_ increases, the corresponding time error does not decrease below 1.06 ns. 

**Figure 15:**
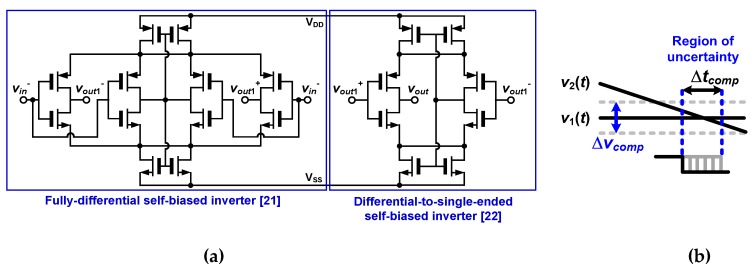
(**a**) Schematic of comparators and (**b**) the uncertainty caused by comparators during time interpolation.

These extra errors would come from the uncertainty of comparator operation in time interpolators. The schematic of comparators and the uncertainty caused by the comparator operation during time interpolation are shown in Figure 15a,b, respectively. The comparators are designed by cascading two self-biased inverters as in [21,22].

As shown in Figure 15b, if there is a voltage-domain uncertainty of Δ*v_comp_* when *v*_1_(*t*) and *v*_2_(*t*) cross each other, Δ*v_comp_* causes a time-domain uncertainty of Δ*t_comp_*, which is given by:

(17) $$\Delta t_{comp}=\Delta v_{comp}\frac{C_{INT}}{I_{INT}}.$$

Therefore, Δ*v_comp_* should be minimized in order to reduce Δ*t_comp_* and hence extra time errors. The finite resolution and noise of the comparator would cause the uncertainty in the voltage domain, which, in turn, is translated into the uncertainty in the time domain.

The resolution of the comparator (Δ*v_min,comp_*), which means the minimum input difference that saturates the output, is expressed as follows [23]:

(18) $$\Delta v_{\min,comp}=\frac{V_{OH}-V_{OL}}{A_{v0}},$$

where *A_v_*_0_ is the open-loop gain of the comparator, *V_OH_* is the output voltage when the output is high, and *V_OL_* is the output voltage when the output is low. *V_OH_* and *V_OL_* should be large and small enough, respectively, so that the following digital logic can distinguish binary states. That is, when the transient behavior of *v*_1_(*t*) and *v*_2_(*t*) is not fast enough or *A_v_*_0_ is not sufficiently large, the comparator suffers from a substantial uncertainty in time domain (Δ*t_comp_*) because it will take relatively longer time for the following digital logic to determine binary states.

Considering that *T_f_*_1_ and *T_f_*_2_ vary within the range from *T_clk_* to 2*T_clk_,* the crossing point of *v*_1_(*t*) and *v*_2_(*t*) in Figure 15b falls within the voltage range from 0.9 V to 1.4 V. In this voltage range, *A_v_*_0_ varies between 41.8 dB and 58.0 dB depending on the voltage level where *v*_1_(*t*) and *v*_2_(*t*) intersect. From *A_v_*_0_ = 41.8 dB and supply voltage of 2.5 V, Δ*v_min,comp_* is about 20.3 mV. For *f_in_* = 128 kHz with *A_T_*_1_ = 15.9 and *A_T_*_2_ = 4.05, Δ*t_comp_* in the first stage caused by Δ*v_min,comp_* is calculated as about 14.6 ns by substituting *C_INT_* = 28.8 pF and *I_INT_* = 40 μA into Equation (17). This Δ*t_comp_* in the first fine stage is divided by *A_T_*_1_ after being quantized by the fine counter. Therefore, the fabricated TDC could not achieve the time error below 0.92 ns.

The noise of comparator would become another source of uncertainty. The input-referred noise voltage of the differential-to-single-ended self-biased inverter in Figure 15a is expressed as follows [24]:

(19) $$\bar{V_{n,inv}{}^{2}\left(f\right)}=\left[\frac{1}{W_{P}L_{P}}+\frac{1}{W_{N}L_{N}}\right]\cdot \frac{2K}{f\cdot C_{OX}}+\frac{8kT\gamma }{g_{m,N}+g_{m,P}},$$

where *W_P_* and *L_P_* are the width and length of the input PMOS transistor, respectively, while *W_N_* and *L_N_* are the width and length of the input NMOS transistor, respectively. *K* is the process-dependent flicker noise constant. *g_m,N_* and *g_m,P_* are the transconductances of the NMOS and PMOS input transistors, respectively. The input-referred noise of the fully differential self-biased inverter is also given by Equation (19).

Since the noise of the first stage is dominant compared to that of the second stage and the 1/f noise can be ignored because of the wide bandwidth of the comparator, the input-referred noise voltage of the comparator can be approximated as follows:

(20) $$\bar{V_{n,comp}{}^{2}\left(f\right)}\approx \frac{8kT\gamma }{g_{m,N}+g_{m,P}}.$$

When *T* = 300 K, *γ* = 1, and noise bandwidth = 100 MHz, the input-referred noise of the comparator is about 1.8 mV_rms_, 180 μV_rms_, and 18 μV_rms_ for *g_m,N_* + *g_m,P_* = 1 μS, *g_m,N_* + *g_m,P_* = 10 μS, *g_m,N_* + *g_m,P_* = 100 μS, respectively. Since our TDC consumes enough current, the uncertainty due to the comparator noise is not significant in our design. However, if the bandwidth of the comparator is very large and *g_m,N_* + *g_m,P_* is small, considerable uncertainties may occur. For *f_in_* = 128 kHz with *A_T_*_1_ = 15.9 and *A_T_*_2_ = 4.05, the voltage-domain uncertainty of 1.8 mV_rms_ causes Δ*t_comp_* = 1.3 ns_rms_, which corresponds to the time error of 0.08 ns_rms_. In particular, it is important to ensure that the value of *g_m,N_* + *g_m,P_* is sufficiently large when the bandwidth of the comparator is wide. 

Since the extra time errors are mainly due to the resolution of comparators, the errors would be mitigated if the comparators based on multi-stage amplifiers are used as in [15]. In such comparators, the optimum number of amplifier stages, *N_OPT_*, is expressed as follows [15]:

(21) $$N_{OPT}\approx 1.1\times \ln\left(\frac{V_{OH}}{\Delta v_{\min,comp}}\right)+0.79.$$

By replacing the two-stage high-gain amplifiers with multiple stages of low-gain amplifiers, the improved Δ*t_comp_* can be obtained as Δ*v_min,comp_* is reduced. 

Moreover, when *A_T_*_2_ increases from 8 to 16, the time error increases rather than decreases. Therefore, another way of improving the extra time error is measuring the quantization error of the first stage without performing time interpolation of the second fine stage. In [19], we employed a chain delay line in the second fine stage instead of the time interpolator, and the results were verified by simulation. Since the mismatches between unit delay cells would occur, the proposed architecture needs to be verified by measurement. 

### 5.5. Performance Summary and Comparison

Table 5 summarizes performances of the presented TDC, together with those of two polar demodulators and two TDCs, reported previously. In comparison with the TDC in [20] that consists of a coarse counter and a single-stage time interpolator with large *A_T_* of 250, our TDC offers lower complexity and shorter conversion time for achieving the same phase resolution, as analyzed in Section 4.3. Compared to the TDC presented in this manuscript, another kind of our TDC in [19] seems to achieve smaller phase error. However, the TDC in [19] has not yet been verified by measurement. Through our future work, the TDC in [19] will be fabricated and measured. The phase error performance of our TDC presented in this paper is competitive when compared with previously reported designs in [15,17].

## 6. Conclusions

A reconfigurable time-to-digital converter (TDC) used to quantize the phase of impedance in electrical impedance spectroscopy (EIS) is introduced in this manuscript and verified through the fabricated IC. This TDC adopts a coarse counter to have a wide input-time range and cascaded time interpolators to improve resolution in the high-frequency analysis without increasing counting clock speed. When the same large interpolation factor is assumed, the cascaded time interpolators have shorter measurement time and smaller chip area than a single-stage time interpolator. The reconfigurable time interpolation factor maintains phase resolution within an appropriate level while providing reasonable measurement time. The fabricated TDC achieves the peak-to-peak phase error of under 0.72° for the input frequency range from 1 kHz to 512 kHz and the peak-to-peak phase error of 2.70° when it covers up to 2.048 MHz, demonstrating competitive performances in comparison with previously reported designs. Two precision improvement methods are also proposed: revision of the comparator, and revision of the second fine stage. As future work, we plan to fabricate the TDC that uses chain delay lines in the second fine stage and compare it with the TDC based on cascaded time interpolators.

## Figures and Tables

**Table 1:** Design specifications of the proposed time-to-digital converter (TDC).

Parameter	Target Level
Application	EIS for biomedical applications
Range of the injected signal frequency (*f_in_*)	1 kHz to 2.048 MHz
Corresponding input-time range for 0° to 90°	0–122 ns (*f_in_* = 2.048 MHz) to 0–250 μs (*f_in_* = 1 kHz)
Phase resolution	>10 bit
Maximum phase error	1°
Corresponding time error	<~1.35 ns (*f_in_* = 2.048 MHz), <~2.8 μs (*f_in_* = 1 kHz)
Reference clock frequency (*f_clk_*)	32.768 MHz

**Table 2:** Assignment of the interpolation factor for different operation modes and values of *f_in._*

Mode	*f_in_* (Hz)	0° to 90°	*f_clk_* (Hz)	Coarse	*A_T_*_1_	*A_T_*_2_
C	2.048M	0~0.1221 μs	32.768M	2 bit	16	16
	1.024M	0~0.2441 μs	32.768M	3 bit	16	16
	512k	0~0.4883 μs	32.768M	4 bit	16	16
	256k	0~0.9766 μs	32.768M	5 bit	16	8
	128k	0~1.9531 μs	32.768M	6 bit	16	4
	64k	0~3.9063 μs	32.768M	7 bit	16	2
B	32k	0~7.8125 μs	32.768M	8 bit	16	N/A
	16k	0~15.625 μs	32.768M	9 bit	8	N/A
	8k	0~31.25 μs	32.768M	10 bit	4	N/A
	4k	0~62.5 μs	32.768M	11 bit	2	N/A
A	2k	0~125 μs	32.768M	12 bit	N/A	N/A
	1k	0~250 μs	16.384M	12 bit	N/A	N/A

**Table 3:** *E_A_*, *E_P_*, and *E_C_* versus *N_f_* when *A_T_* is set by the capacitance ratio.

*A_T_*	*N_f_*	*E_A_*	*E_P_*	*E_C_*	*E_A_·E_P_·E_C_*
256	2	7.6	8	0.5	30.4
256	4	12.9	16	0.25	51.6
256	8	10.7	16	0.125	21.4

**Table 4:** Peak-to-peak phase error and corresponding time error in mode C.

*f_in_* (Hz)	*A_T_*_1_	*A_T_*_2_	Target Phase Error (°)	Peak-to-Peak Phase Error (°)	Corresponding Time Error (ns)
64k	16	2	0.022	0.037	1.61
128k	16	4	0.022	0.049	1.06
256k	16	8	0.022	0.178	1.93
512k	16	16	0.022	0.721	3.91
1.024M	16	16	0.088	1.194	3.29
2.048M	16	16	0.088	2.696	3.66

**Table 5:** Performance summary and comparison.

	This Work	*IEEE Sensors J.’* 2013 [15]	IEEE MWSCAS’ 2013 [17]	*IEEE TNS.’* 2006 [20]	IEEE MWSCAS’ 2017 [19]
Tech.	0.25 μm	0.35 μm	0.35 μm	0.35 μm	0.18 μm
Application	EIS	EIS	EIS	N/A	EIS
Implementation scope	TDC	Polar demodulator	Polar demodulator	TDC	TDC
Architecture	Counter + Cascaded time interpolator	TVC with ADC	Counter	Counter + time stretchers	Counter+ time stretcher + chain-delay-line
*f_in_*	1 kHz– 2.048 MHz	0.1 kHz– 100 kHz	0.1 kHz– 10 MHz	N/A	1 kHz– 2.048 MHz
*f_clk_*	32.768 MHz	No use	3.33 GHz	80 MHz	32.768 MHz
Power	7.5 mW	21 mW	*28 mW	0.75 mW	**2.4 mW**
Supply	2.5 V	2.5 V	1.8 V	3 V–4 V	1.8 V
Area	0.41 mm^2^	*0.40 mm^2^	*0.40 mm^2^	0.23 mm^2^	0.35 mm^2^
Time resolution	N/A	N/A	300 ps	50 ps	103 ps–244 ns
Phase error	<0.72° (@512 kHz) <2.70° (@2.048 MHz)	<3.95°	<2.2°	N/A	<0.088°
Remarks	Meas.	Meas.	Meas.	Meas.	Sim.

* Total power consumption or size of entire polar demodulator for EIS system.
